# Medical imaging utilization and associated radiation exposure in children with down syndrome

**DOI:** 10.1371/journal.pone.0289957

**Published:** 2023-09-06

**Authors:** Emily C. Marlow, Jonathan M. Ducore, Marilyn L. Kwan, Erin J. A. Bowles, Robert T. Greenlee, Jason D. Pole, Alanna K. Rahm, Natasha K. Stout, Sheila Weinmann, Rebecca Smith-Bindman, Diana L. Miglioretti

**Affiliations:** 1 Department of Surveillance & Health Equity Science, American Cancer Society, Kennesaw, Georgia, United States of America; 2 Department of Pediatrics, University of California, Davis, California, United States of America; 3 Division of Research, Kaiser Permanente Northern California, Oakland, California, United States of America; 4 Kaiser Permanente Washington Health Research Institute, Kaiser Permanente Washington, Seattle, Washington, United States of America; 5 Marshfield Clinic Research Institute, Marshfield Clinic Health System, Marshfield, Wisconsin, United States of America; 6 Centre for Health Service Research, University of Queensland, Brisbane, Australia; 7 Dalla Lana School of Public Health University of Toronto, Toronto, Canada; 8 ICES Toronto, Ontario, Canada; 9 Department of Genomic Health, Geisinger, Danville, PA, United States of America; 10 Department of Population Medicine, Harvard Medical School, Boston, Massachusetts, United States of America; 11 Harvard Pilgrim Health Care Institute, Boston, Massachusetts, United States of America; 12 Kaiser Permanente Center for Health Research, Portland, Oregon, United States of America; 13 Center for Integrated Health Care Research, Kaiser Permanente Hawaii, Honolulu, Hawaii, United States of America; 14 Department of Biostatistics and Epidemiology, University of California, San Francisco, California, United States of America; 15 Department of Obstetrics, Gynecology, and Reproductive Medicine, University of California, San Francisco, California, United States of America; 16 Philip R. Lee Institute for Health Policy Studies, University of California, San Francisco, California, United States of America; 17 Department of Public Health Sciences, University of California, Davis, California, United States of America; University Hospital Dubrava, University of Zagreb School of Medicine, CROATIA

## Abstract

**Objective:**

To evaluate the frequency of medical imaging or estimated associated radiation exposure in children with Down syndrome.

**Methods:**

This retrospective cohort study included 4,348,226 children enrolled in six U.S. integrated healthcare systems from 1996–2016, 3,095 of whom were diagnosed with Down syndrome. We calculated imaging rates per 100 person years and associated red bone marrow dose (mGy). Relative rates (RR) of imaging in children with versus without Down syndrome were estimated using overdispersed Poisson regression.

**Results:**

Compared to other children, children with Down syndrome received imaging using ionizing radiation at 9.5 times (95% confidence interval[CI] = 8.2–10.9) the rate when age <1 year and 2.3 times (95% CI = 2.0–2.5) between ages 1–18 years. Imaging rates by modality in children <1 year with Down syndrome compared with other children were: computed tomography (6.6 vs. 2.0, RR = 3.1[95%CI = 1.8–5.1]), fluoroscopy (37.1 vs. 3.1, RR 11.9[95%CI 9.5–14.8]), angiography (7.6 vs. 0.2, RR = 35.8[95%CI = 20.6–62.2]), nuclear medicine (6.0 vs. 0.6, RR = 8.2[95% CI = 5.3–12.7]), radiography (419.7 vs. 36.9, RR = 11.3[95%CI = 10.0–12.9], magnetic resonance imaging(7.3 vs. 1.5, RR = 4.2[95% CI = 3.1–5.8]), and ultrasound (231.2 vs. 16.4, RR = 12.6[95% CI = 9.9–15.9]). Mean cumulative red bone marrow dose from imaging over a mean of 4.2 years was 2-fold higher in children with Down syndrome compared with other children (4.7 vs. 1.9mGy).

**Conclusions:**

Children with Down syndrome experienced more medical imaging and higher radiation exposure than other children, especially at young ages when they are more vulnerable to radiation. Clinicians should consider incorporating strategic management decisions when imaging this high-risk population.

## Introduction

Medical imaging utilization rates in children have increased in the past two decades, most notably for computed tomography (CT) which delivers higher doses of ionizing radiation in comparison with other modalities [1,2]. Children with genetic syndromes that predispose them to cancer may undergo more frequent imaging for diagnosis and monitoring of symptoms related to co-morbidities, especially during early life [3,4]. The most common childhood condition with cancer susceptibility is Down syndrome, affecting 1.4 in 1,000 live births [5]. Although the magnitude of imaging utilization in children with Down syndrome has not been studied previously, they are expected to undergo higher rates of imaging due to co-morbid conditions, including congenital heart disease [6,7] and central nervous system and musculoskeletal abnormalities [8–11]. Children with Down syndrome are at higher risk of developing acute myeloid leukemia, acute lymphoid leukemia, and acute megakaryoblastic leukemia [12]. Many studies have shown that children exposed to ionizing radiation within the dose ranges commonly used for medical imaging have an increased risk of leukemia [13–15]. Children with Down syndrome have known defects in DNA repair and thus may be more susceptible to cancer from radiation induced DNA damage. In one of the largest and most comprehensive studies to date, we examined rates of medical imaging and associated ionizing radiation exposure in children with Down syndrome compared with other children.

## Materials and methods

### Study population

This retrospective cohort study included all children enrolled for at least six months in one of six U.S. integrated healthcare systems between January 1, 1996 and December 30, 2016 (N = 4,348,226). The participating healthcare systems are members of the Health Care Systems Research Network (HCSRN) [16] and include Kaiser Permanente (KP) Hawaii, KP Northern California, KP Northwest (Oregon/Southwest Washington), and KP Washington; Harvard Pilgrim Health Care (Boston, Massachusetts); and Marshfield Clinic Health System (Wisconsin).

Children were followed from the start date of their enrollment in the healthcare system until censoring at age 19 years, disenrollment from the healthcare system, death, six months prior to a cancer diagnosis, or end of follow up (December 31, 2016). We conservatively excluded imaging performed within six months prior to a cancer diagnosis to avoid including imaging used to diagnose the cancer [17,18]. Children could contribute additional enrollment periods after a disenrollment. The following institutional review boards approved this data-only study with a waiver of informed consent: Harvard Pilgrim Health Care, KP Hawaii, KP Northern California, KP Northwest, KP Washington, Marshfield Clinic, University of California Davis, University of California San Francisco, and University of Toronto Health Sciences.

### Identifying Down syndrome

Down syndrome was identified using International Classification of Diseases (ICD) diagnosis 9^th^ edition code 758.0 and 10^th^ edition codes Q90.0, Q90.1, Q90.2, or Q90.9. Down syndrome diagnoses were obtained from clinical and administrative data including electronic health records through the Virtual Data Warehouse (VDW) [16], a set of data standards and programs applied at each U.S. site to their raw administrative and billing data, ensuring data compatibility and harmonization for multi-site studies.

No validated approach exists for defining a child with Down syndrome using electronic healthcare records [19]. Most children in our cohort with Down syndrome diagnostic codes had codes on many different days during enrollment (mean = 59.3 [standard deviation(SD) = 84.0], median = 28 days). Based on a small validation study of N = 48 children with codes on <5 days [12], we considered children who had Down syndrome diagnosis codes on at least three unique days or had Down syndrome confirmed during detailed chart review to have Down syndrome, whereas children with Down syndrome codes identified on only one or two days were excluded from further analysis to avoid potential information bias.

### Identifying imaging examinations

We ascertained imaging examinations from electronic health records. The imaging examinations were coded using Current Procedural Terminology [19]; International Classification of Diseases, Ninth Revision, Clinical Modification[20]; International Statistical Classification of Diseases, Tenth Revision, Clinical Modification [21]; and Healthcare Common Procedure Coding System [16] billing codes, including modifiers for the technical, physician, or global components. Examinations were included irrespective of the physician specialty billing for the study. Clinical reason for examination order was unknown in the database. Some billing codes changed over time, and all codes were mapped to an anatomic area and imaging modality to ensure consistency over time, updating a previously used map [1]. Imaging modalities were classified as angiography, computed tomography (CT), fluoroscopy, magnetic resonance imaging (MRI), ultrasound, nuclear medicine or radiography. MRI and ultrasound do not use ionizing radiation. Anatomic areas were classified as abdomen (including any imaging of the abdomen and/or pelvis), chest/cardiac, extremity, head and brain, neck (including cervical spine), and thoracic and lumbar spine. Exams where the anatomic area was not specified were excluded if there was another examination of the same modality with a known anatomic area on the same day. To avoid potential duplicate exam counting, we restricted imaging to a maximum of one exam per modality and anatomic area per day.

### Substudy with matched design for radiation dose estimation

To estimate cumulative radiation dose for children with Down syndrome and compare with other children, a matched subset of the cohort was selected to control for several potential confounding factors and follow-up time. We created strata based on the child’s study site, sex, age at study entry, and year of study entry. In the first matching phase, 2,564 children with Down syndrome were matched, using random sampling without replacement, to 10 children without Down syndrome from the same strata who had a follow-up time at least as long. The follow-up time for all children from the same matched set was set to that of the child with Down syndrome. A total of 531 children with Down syndrome did not match to at least 10 children without Down syndrome with adequate follow-up time in the stratum. Thus, we implemented a second matching phase, matching these 531 children with Down syndrome to the 10 children without Down syndrome with the longest follow-up time from the stratum. We truncated the follow-up time for all children in the matched set to shortest follow-up time in the set. We combined the matched sets from the first and second matching phases to create the final matched subset. For this matched substudy, we only included examinations that occurred within the newly defined follow-up time.

We chose absorbed red bone marrow dose as our primary outcome of radiation exposure, because leukemia risk is most closely associated with red marrow dose [22], and children with Down syndrome are at increased risk of leukemia [12]. We created a detailed map of estimated red bone marrow dose associated with each imaging exam that uses ionizing radiation, stratified by modality, anatomic area, age, and sex of the patient using a combination of doses observed in our cohort (for CT), detailed Monte Carlo modeling (for angiography, fluoroscopy, and radiography), and the published literature (nuclear medicine). More details have been published previously [23]. We estimated cumulative radiation exposure by adding a child’s absorbed red bone marrow dose from each exam conducted during the follow-up period.

### Statistical methods

Descriptive characteristics were calculated in the cohort of unique children with and without Down syndrome. Imaging rates (per 100 person-years) were calculated for children with and without Down syndrome by use of ionizing radiation and modality. Imaging rates by calendar year and age (years) were graphed for children with and without Down syndrome, stratified by imaging modality and anatomic area using a three-year moving average. The distribution of number of exams per child were examined by Down syndrome status, age group, and imaging modality. The average cumulative estimated red marrow dose per child contributed by each modality was compared between children with and without Down syndrome within the matched substudy sample.

We used Poisson regression accounting for overdispersion to estimate the association (relative rates [RRs]) between Down syndrome and imaging utilization by birth year group (1996–2000, 2001–2005, 2006–2010, 2011–2016) and calendar year (1996–2000, 2001–2005, 2006–2010, 2011–2016), adjusting for sex (male, female), race (White, Black, Asian/Pacific Islander, Unknown/Other), ethnicity (Hispanic, Non-Hispanic, Unknown), Medicaid status (yes, no), and healthcare system. We estimated standard errors and 95% confidence intervals (CI) using generalized estimating equations with a working independence correlation structure to account for multiple observations on the same child. Each child contributed one observation for each year of age and calendar year enrolled. Enrollment time on the log-scale was included as an offset term. Given the large number of observations, Proc Genmod in SAS could not support the full dataset, so we divided the data into two datasets and combined the estimates by calculating the weighted average on the log-scale, weighting inversely by the standard error.^18^ We explored effect modification of the association between Down syndrome and imaging utilization by age groups, sex, and calendar year by including interaction terms between Down syndrome status and these factors.

## Results

Down syndrome was diagnosed in 3,095 of 4,348,226 children (prevalence of Down syndrome 0.7 per 1,000 children; Table 1). Children with Down syndrome had longer follow-up time than children without Down syndrome (median = 3.91 [Q1 = 1.44, Q3 = 7.84] vs. median = 2.75 [Q1 = 1.07, Q3 = 6.07], person-years), and were more likely to enter the study at younger ages than other children (age <1: 48% vs. 31%). The prevalence of Down syndrome was higher in children <1 year old at the start of the follow-up period (1.1 per 1,000 children) compared with older children (0.4/1,000). It was also higher in White (1.1/1,000) compared with Black and Asian children (both 0.8/1,000), and higher in Hispanic (1.1/1,000) compared with non-Hispanic children (0.7/1,000).

**Table 1:** Characteristics of children with and without Down syndrome enrolled in six U.S. healthcare systems between 1996–2016.

	Children with Down Syndrome		Children without Down Syndrome		Prevalence of Down Syndrome (per 1,000 children)
** **	***N***	**(%)** *†*	***N***	***(%)†***	
**Total**	3,095	0.1%	4,345,131	99.9%	0.7
**Person-years of follow-up, Median (Q1, Q3)**	3.91 (1.44, 7.84)			2.75 (1.07, 6.07)	
**Sites**					
Site 1	84	2.7%	162,250	***3*.*7%***	0.5
Site 2	92	3.0%	111,926	2.6%	0.8
Site 3	235	7.6%	382,345	8.8%	0.6
Site 4	280	9.0%	406,688	9.4%	0.7
Site 5	601	19.4%	762,312	17.5%	0.8
Site 6	1,803	58.3%	2,519,610	58.0%	0.7
**Age at Study Entry, years**					
<1	1,485	48.0%	1,328,361	30.6%	1.1
1–4	590	19.1%	901,119	20.7%	0.7
5–9	539	17.4%	958,404	22.1%	0.6
10–14	346	11.2%	793,759	18.3%	0.4
15–18	135	4.4%	363,488	8.4%	0.4
**Age at End of Follow Up, years**					
<1	334	10.8%	300,334	6.9%	1.1
1–4	950	30.7%	1,116,683	25.7%	0.9
5–9	805	26.0%	1,128,252	26.0%	0.7
10–14	600	19.4%	1,005,658	23.1%	0.6
15–18	406	13.1%	794,204	***18*.*3%***	0.5
**Calendar Year at Study Entry**					
1996–2000	1032	33.3%	1,796,452	41.3%	0.6
2001–2005	616	19.9%	826,899	19.0%	0.7
2006–2010	666	21.5%	805,996	18.5%	0.8
2011–2016	781	25.2%	915,784	21.1%	0.9
**Calendar Year at End of Follow Up**					
1996–2000	703	22.7%	1,338,082	30.8%	0.5
2001–2005	456	14.7%	766,378	17.6%	0.6
2006–2010	503	16.3%	685,522	15.8%	0.7
2011–2016	1433	46.3%	1,555,149	35.8%	0.9
**Sex**					
Male	1439	46.5%	2,128,117	49.0%	0.7
Female	1656	53.5%	2,217,014	51.0%	0.7
**Race**					
White	1469	72.1%	1,302,437	64.0%	1.1
Black	189	9.3%	240,968	11.8%	0.8
Asian or Pacific Islander	380	18.6%	490,956	***24*.*1%***	0.8
Unknown or Other	1057	(34.2%)	2,310,770	(53.2%)	0.5
**Ethnicity**					
Hispanic	682	26.0%	593,332	17.8%	1.1
Non-Hispanic	1937	74.0%	2,734,666	82.2%	0.7
Unknown	476	(15.4%)	1,017,133	(23.4%)	0.5
**Medicaid**					
Yes	240	7.8%	359,967	8.3%	0.7
No	2855	92.2%	3,985,164	91.7%	0.7

*Q1 = first quartile; Q3 = third quartile; †Column percentages exclude “Unknown or Other”.

**Fig 1:**
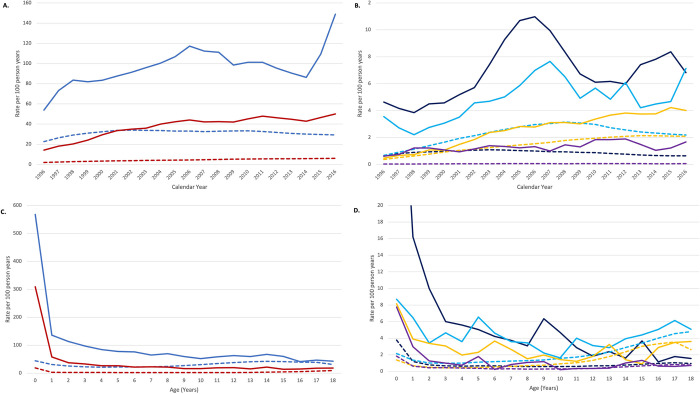
Imaging rates per 100 person years by calendar year (A-B) and age (C-D), by imaging modality and Down syndrome diagnosis. Graphs show a three-year moving average.

Children with Down syndrome had higher imaging rates than other children in all calendar years, and imaging rates increased steadily over time for most modalities (Fig 1A and 1B). For example, among children with Down syndrome from 1996 to 2016, radiography increased from 54 to 149 tests per 100 person years, ultrasound ***from*** 14 to 50, fluoroscopy from 5 to 7, CT from 4 to 7 and MRI from 0.5 to 4. The absolute difference in imaging rates between children with and without Down syndrome increased, in general, over time due to the rising rates of imaging in children with Down syndrome and declining rates of imaging in children without Down syndrome. In children with Down syndrome, imaging utilization rates declined with increasing age for radiography, ultrasound, fluoroscopy, angiography, and nuclear medicine; imaging rates declined from ages 0–9 years and then increased starting around age 10 or 11 years for CT and MRI (Fig 1C and 1D). Imaging rates were particularly high in children with Down syndrome <1 year of age (Fig 1C and 1D). Imaging rates in children without Down syndrome were lower than those with Down syndrome at all ages and increased with age after age 3 years.

Imaging rates in children with and without Down syndrome are reported separately by age (<1 vs 1–18 years) and imaging modality in Table 2. Within the first year of life, children with Down syndrome received 9.5 times (95% CI = 8.2–10.9) the rate of medical imaging with ionizing radiation compared with children without Down syndrome, 477.0 vs. 42.7 tests per 100 person years (Table 2). In children ages 1–18 years, the RR of imaging with ionizing radiation in children with Down syndrome compared with those without was more moderate (156.3 vs. 68.5 tests per 100 person years; RR = 2.3, 95% CI = 2.0–2.5). In children age <1 year, children with Down syndrome received 3.1 times (95% CI = 1.8–5.1) the rate of CT imaging than children without Down syndrome, 6.6 vs. 2.0 tests per 100 person years. The RR of imaging in children age <1 year with Down syndrome compared with those without was even higher in other imaging modalities, including nuclear medicine (RR = 8.2, 95% CI = 5.3–12.7), radiography (RR = 11.3, 95% CI = 10.0–12.9), fluoroscopy (RR = 11.9, 95% CI = 9.5–14.8), and angiography (RR = 35.8, 95% CI = 20.6–62.2).

**Table 2:** Imaging rates per 100 person-years by modality for children with and without Down Syndrome at age <1 year and between 1–18 years. Relative rates (RR) by modality and age group compare rates in children with vs. without Down syndrome.

	**Children with Down Syndrome** **(N = 1,226 person years)**			**Children without Down Syndrome (N = 1,035,882 person years)**				
**Age < 1 year**								
**Tests with ionizing radiation**	**No. Exams**	**Rate per 100py**	**95% CI**	**No. Exams**	**Rate per 100py**	**95% CI**	**RR**	**95% CI**
All ionizing radiation imaging	5,848	477.0	(464.9, 489.3)	442,756	42.7	(42.6, 42.9)	9.5	(8.2, 10.9)
Computed Tomography	81	6.6	(5.3, 8.2)	21,154	2.0	(2.0, 2.1)	3.1	(1.8, 5.1)
Radiography	5,146	419.7	(408.3, 431.4)	382,108	36.9	(36.8, 37.0)	11.3	(10.0, 12.9)
Fluorography	455	37.1	(33.8, 40.7)	31,684	3.1	(3.0, 3.1)	11.9	(9.5, 14.8)
Angiography	93	7.6	(6.1, 9.3)	1,856	0.2	(0.2, 0.2)	35.8	(20.6, 62.2)
Nuclear Medicine	73	6.0	(4.7, 7.5)	5,954	0.6	(0.6, 0.6)	8.2	(5.3, 12.7)
**Tests without ionizing radiation**	**No. Exams**	**Rate per 100py**	**95% CI**	**No. Exams**	**Rate per 100py**	**95% CI**	**RR**	**95% CI**
MRI	90	7.3	(72.9, 73.9)	15,475	1.5	(1.5, 1.5)	4.2	(3.1, 5.)
Ultrasound	2,835	231.2	(231.0, 231.5)	170,209	16.4	(16.4, 16.5)	12.6	(9.9, 15.9)
	**Down Syndrome (N = 9,687 person years)**			**No Down Syndrome (N = 11,074,005 person years)**				
**Age 1–18 years**								
**Tests with ionizing radiation**	**No. Exams**	**Rate per 100py**	**95% CI**	**No. Exams**	**Rate per 100py**	**95% CI**	**RR**	**95% CI**
All ionizing radiation imaging	15,143	156.3	(153.8, 158.8)	7,583,330	68.5	(68.4, 68.5)	2.3	(2.0, 2.5)
Computed Tomography	830	8.6	(8.0, 9.2)	480,222	4.3	(4.3, 4.3)	2.0	(1.7, 2.4)
Radiography	13,132	135.6	(133.3, 137.9)	6,881,011	62.1	(62.1, 62.2)	2.2	(2.0, 2.4)
Fluorography	808	8.3	(7.8, 8.9)	160,911	1.5	(1.4, 1.5)	4.7	(3.6, 6.1)
Angiography	205	2.1	(1.8, 2.4)	11,320	0.01	(0.01, 0.02)	18.0	(11.6, 28.1)
Nuclear Medicine	168	1.7	(1.5, 2.0)	49,866	0.5	(0.4, 0.5)	3.5	(2.2, 5.4)
**Tests without ionizing radiation**	**No. Exams**	**Rate per 100py**	**95% CI**	**No. Exams**	**Rate per 100py**	**95% CI**	**RR**	**95% CI**
MRI	523	5.4	(4.9, 5.9)	330,181	3.0	(3.0, 3.0)	1.7	(1.0, 2.9)
Ultrasound	3,986	41.1	(39.9, 42.4)	794,426	7.2	(7.2, 7.2)	3.5	(2.2, 5.4)

py = person-years; RR = unadjusted relative rate; CI = confidence interval.

**Fig 2:**
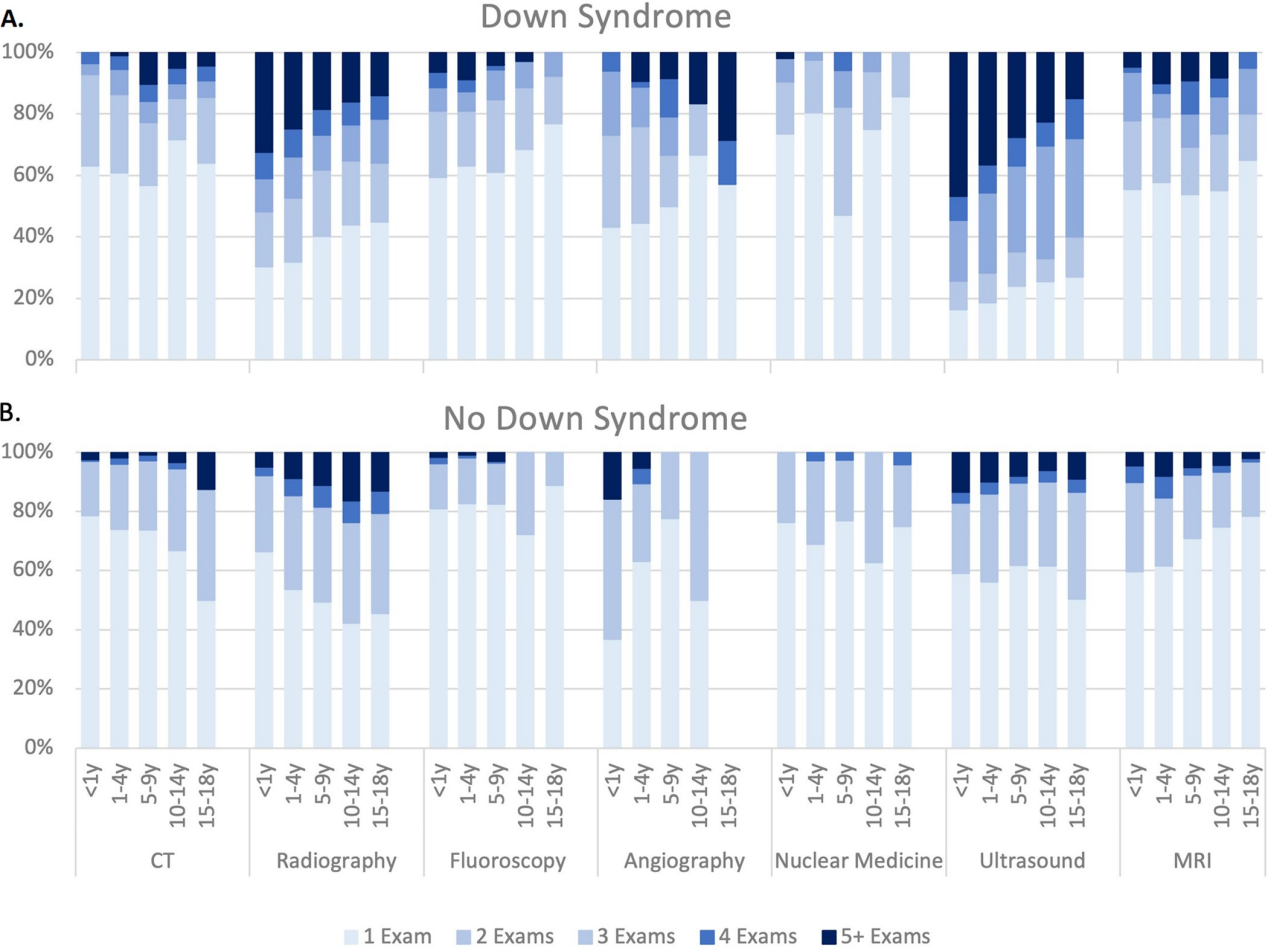
Distribution of number of imaging examinations in children with at least one exam by age group and modality. A = children with Down syndrome and B = children without Down syndrome.

Children with Down syndrome were also more likely to undergo multiple imaging exams of the same modality (Fig 2A and 2B). Multiple radiography exams were particularly common in younger children with Down syndrome; in children with at least one radiography examination during the first year of life, 32% of children with vs. 5% of children without Down syndrome underwent five or more radiography exams. In children ages 1–4 years with at least one exam, 25% of children with vs. 9% of children without Down syndrome underwent at least five radiography exams. Children with Down syndrome also underwent more ultrasound exams. Among children with at least one ultrasound examination during the first year of life, 84% of children with vs. 41% of children without Down syndrome underwent at least two ultrasound exams and 47% versus 13% underwent at least five.

**Fig 3:**
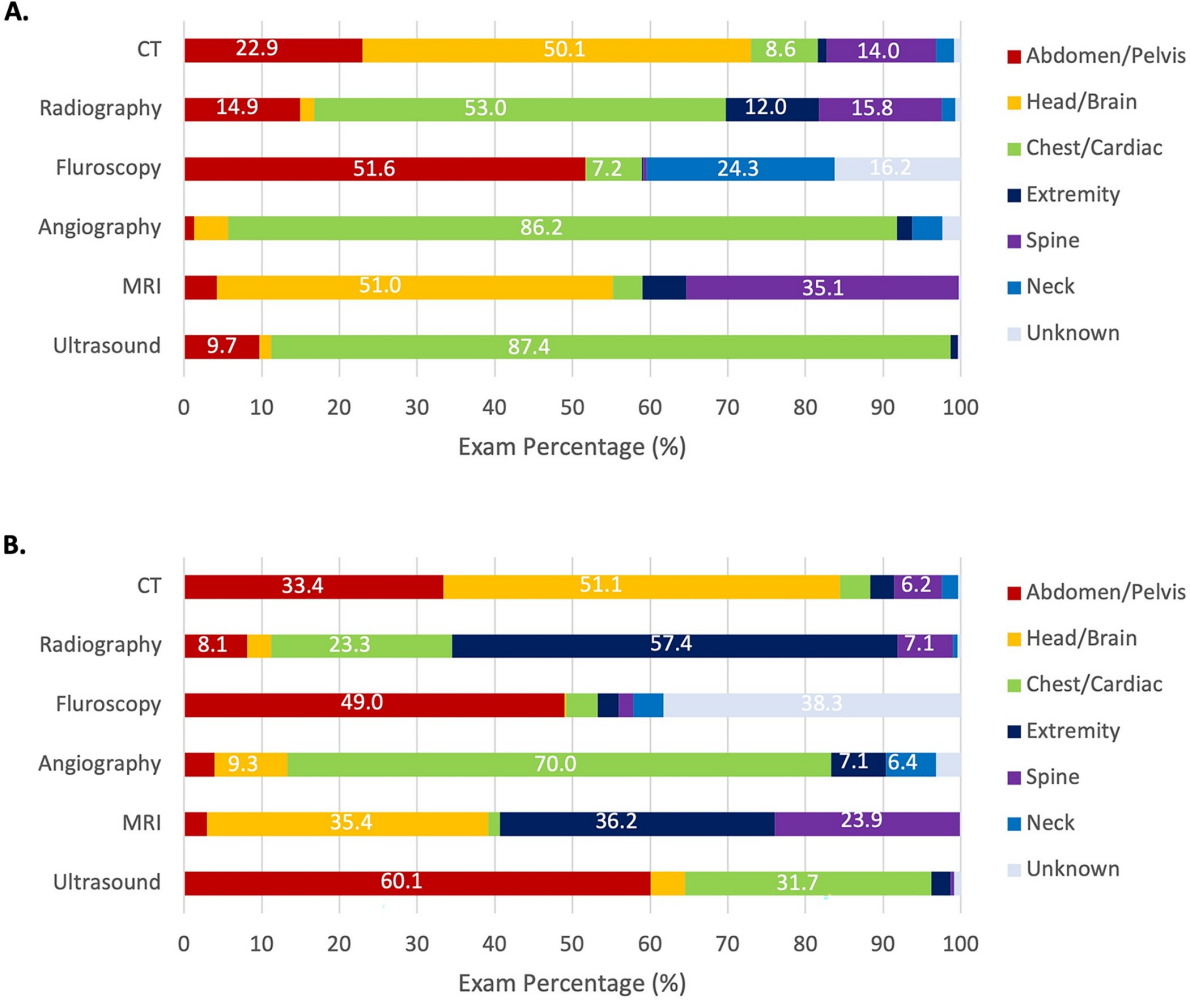
Distribution (percentages) of anatomic areas imaged for each modality in children with (A) and without (B) Down syndrome. The values of percentages larger than 6% are shown.

In Fig 3, we examined the distribution of anatomic region imaged within each modality. For children with Down syndrome, 9.7% of ultrasound exams and 22.9% of CT exams were conducted on the abdomen and/or pelvis vs. 60.1% and 33.4% in children without Down syndrome. For CT exams, 50.1% and 51.1% of exams were conducted in the head/brain region on children with and without Down syndrome, respectively. For radiography exams, children with Down syndrome had a higher percentage of chest/cardiac exams and lower percentage of extremity exams than other children.

Adjusted RRs of imaging were evaluated in children with vs. without Down syndrome (Table 3). The relative increased use of imaging was most pronounced in the youngest children, e.g., children with Down syndrome <1 year had 3.1 times the rate of CT exams (95% CI = 1.8–5.1) and 35.8 times the rate of angiography (95%CI = 20.6–62.2) than children without Down syndrome. RRs were similar over time for CT, radiography, and MRI; RRs declined in most recent time period for ultrasound and became more pronounced in the most recent time period for fluoroscopy, angiography, and nuclear medicine (Table 3).

**Table 3:** Relative rates of imaging (95% confidence intervals) comparing children with vs. without Down syndrome for each modality by age, calendar year, sex, and Medicaid status, adjusted for the other variables listed as well as race, ethnicity, and healthcare system.

	Computed Tomography	Radiography	Fluoroscopy	Angiography	Nuclear Medicine	Ultrasound	MRI
Age, years	RR (95% CI)	RR (95% CI)	RR (95% CI)	RR (95% CI)	RR (95% CI)	RR (95% CI)	RR (95% CI)
<1	3.1 (1.8, 5.1)	11.3 (10.0, 12.9)	11.9 (9.5, 14.8)	35.8 (20.6, 62.2)	8.2 (7.0, 46.0)	12.6 (9.9, 15.9)	4.2 (3.1, 5.8)
1–4	3.2 (2.5, 4.0)	4.3 (3.9, 4.7)	12.2 (9.6, 15.6)	27.9 (13.1, 59.6)	4.3 (2.8, 6.6)	11.1 (8.1, 15.1)	4.9 (3.6, 6.7)
5–9	3.3 (2.6, 4.3)	2.9 (2.5, 3.2)	5.8 (4.2, 8.0)	27.2 (16.3, 45.4)	4.0 (2.3, 6.8)	7.8 (6.0, 10.2)	3.0 (2.2, 4.0)
10–14	1.7 (1.3, 2.2)	1.5 (1.3, 1.7)	3.9 (2.8, 5.3)	11.5 (5.0, 26.3)	2.5 (1.4, 4.2)	5.6 (3.8, 8.3)	1.1 (0.6, 2.2)
15–18	1.4 (1.1, 1.9)	1.4 (1.2, 1.6)	2.0 (1.4, 2.9)	12.3 (6.1, 24.5)	2.5 (1.0, 6.25)	2.0 (1.7, 2.4)	0.9 (0.4, 2.1)
**Calendar Year**							
1996–2000	2.7 (2.0, 3.6)	3.0 (2.1, 4.3)	5.4 (3.0, 9.5)	25.0 (12.0, 52.1)	3.3 (1.8, 5.9)	7.2 (6.2, 8.3)	2.0 (0.9, 4.6)
2001–2005	1.9 (1.4, 2.6)	3.0 (2.6, 3.3)	4.9 (3.7, 6.6)	16.3 (9.8, 27.0)	3.3 (2.0, 5.5)	8.3 (7.2, 9.6)	2.4 (1.4, 4.1)
2006–2010	2.1 (1.7, 2.8)	3.3 (2.9, 3.7)	5.7 (4.3, 7.6)	16.7 (11.4, 24.5)	3.8 (2.4, 6.0)	6.5 (4.8, 8.9)	2.2 (1.7, 2.9)
2011–2016	2.7 (2.2, 3.3)	3.2 (2.6, 3.8)	7.4 (5.6, 9.7)	26.9 (11.4, 63.5)	5.6 (3.1, 10.1)	4.8 (2.0, 11.8)	2.5 (1.9, 3.1)
**Sex**							
Male	2.3 (1.9, 2.8)	2.8 (2.6, 3.1)	5.7 (4.5, 7.2)	17.0 (8.9, 32.4)	4.6 (2.6, 8.2)	7.8 (6.6, 9.2)	2.2 (1.7, 2.9)
Female	2.5 (2.0, 3.0)	3.4 (3.1, 3.9)	5.9 (4.5, 7.6)	24.4 (14.2, 41.9)	3.3 (2.1, 5.0)	5.5 (3.9, 7.9)	2.4 (1.7, 3.6)
**Medicaid Status**							
Yes	2.6 (2.3, 3.0)	3.1 (2.9, 3.3)	5.4 (3.7, 7.8)	23.3 (17.9, 30.2)	5.0 (4.0, 6.3)	7.8 (6.6, 9.3)	2.5 (2.1, 2.9)
No	2.2 (1.6, 2.9)	3.1 (2.6, 3.7)	6.3 (4.2, 9.3)	17.9 (7.0, 46.0)	3.0 (1.4, 6.3)	5.5 (3.9, 7.9)	2.9 (2.1, 4.1)

RR = relative rate; CI = confidence interval.

**Fig 4:**
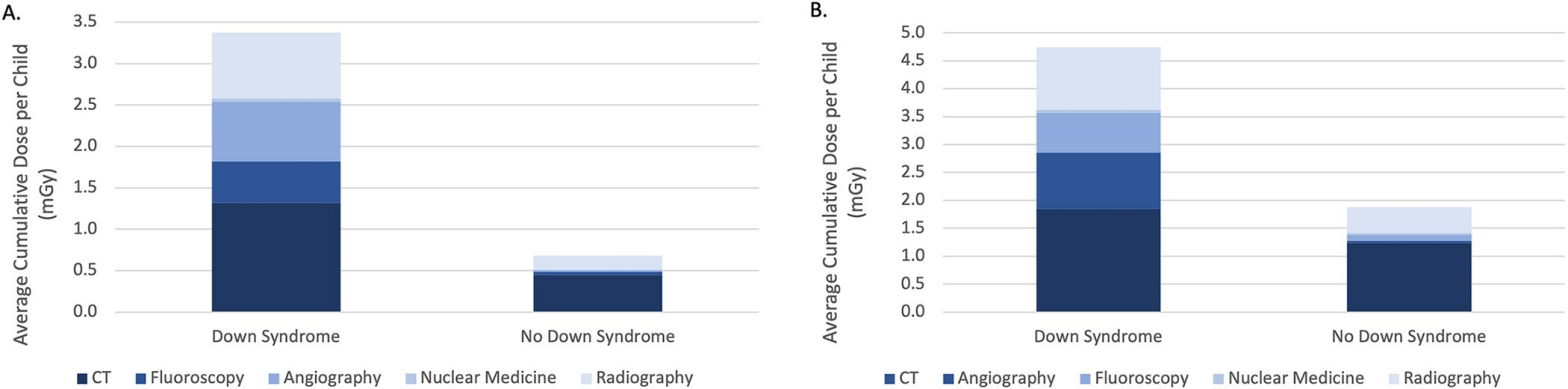
Average cumulative radiation exposure per child contributed by each modality in children with Down syndrome and 10 matched children without Down syndrome. A = average exposure levels for all children (7.0 mean years of follow-up). B = average exposure levels in children exposed to any imaging (7.2 mean years of follow-up).

The mean cumulative estimated red bone marrow dose per child from each modality in children with and without Down syndrome in the matched substudy is shown in Fig 4A and 4B. Among all children, children with Down syndrome had more than double the mean cumulative red bone marrow dose than children without Down syndrome (4.7 vs. 1.9 mGy; 6.99 mean years of follow-up, Fig 4A). Among children who had at least one imaging exam with radiation, children with vs. without Down syndrome had five times the mean cumulative red bone marrow dose per child (3.4 vs. 0.68 mGy; 7.2 mean years of follow-up, Fig 4B).

## Discussion

In this contemporary cohort of over 4 million children in the U.S., those with Down syndrome underwent significantly more medical imaging compared with children without Down syndrome. Imaging rates were highest within the first year of life in both children with and without Down syndrome, but children with Down syndrome had almost 10-times the rate of imaging examinations that use ionizing radiation than children without Down syndrome. Children with Down syndrome were also more likely to have multiple exams than children without Down syndrome. The greater exposure to imaging using ionizing radiation, and greater number of exposures for each type of exam, resulted in children with Down syndrome receiving more than double the mean estimated cumulative red bone marrow dose per child compared to other children, and more than five times the radiation dose when restricting to children with any imaging using ionizing radiation.

In general, imaging in children using ionizing radiation has decreased in the last decade [1]. However, we did not observe the same decline in children with Down syndrome, with a persistent rise in imaging using ionizing radiation, including fluoroscopy, CT, nuclear medicine, and angiography. It is unclear why use of all modalities has risen in children with Down syndrome, while children without Down syndrome only observed increases in MRI and decreases in imaging with ionizing radiation. Radiography of the extremities is the only type of imaging that was less common in children with Down syndrome, potentially because they are less likely to participate in sports than other children.

More imaging use in children with Down syndrome is expected given their concomitant medical diagnoses and anomalies [9–11]. Exposure to radiation in childhood entails a three- to five-fold greater risk of cancer compared to similar exposures in adults [24–27]. Therefore, these markedly elevated exposures to ionizing radiation from medical imaging in children with Down syndrome should be reduced whenever possible, possibly by using MRI or ultrasound which do not use ionizing radiation. Children with Down syndrome may be especially sensitive to developing cancer from ionizing radiation because of their poor DNA repair mechanisms [28]. It is unknown whether this greater use of imaging may contribute to their elevated risk of leukemia [12]; however, further study is currently underway [23].

Campaigns like Image Gently have helped raise awareness of the need to reduce the radiation used in pediatric radiology [29–32]. Surveys have indicated a willingness of radiologists to adopt lower dose protocols [32]. However, there has been less focus on reducing pediatric imaging utilization rates. Imaging utilization with ionizing radiation has not shown large decreases over time and even seems to be increasing in recent years [1]. A recent study suggested that CT exams for mild traumatic head injuries in children could be reduced as much as 29% following the PECARN rule to assess medical need [33]. Though head CT usage in children with Down syndrome is not limited to mild trauma, implementing similar strategies from the PECARN rule is an opportunity to help reduce general CT examination in these high risk children.

There are tradeoffs when it comes to reducing imaging, especially in populations that experience multiple medical abnormalities. Multiple factors are considered by medical professionals when choosing diagnostic tests, such as patient tolerance and result speed. Use of imaging should be justified without compromising care, with optimization of radiation exposure when imaging is medically necessary [34]. Future research should determine the value of imaging in Down syndrome with the goal of reducing imaging if it does not directly impact medical decision making. Our results suggests that imaging rates are very high in children with Down syndrome, and therefore, our results may be used to justify the need for explicit guidelines to help appropriately image these children; the potential benefits of imaging need to be balanced against potential harms including elevated cancer risk from medical imaging with ionizing radiation. Because of neurocognitive challenges, children with Down syndrome may be less able to localize their pain, and this may result in more imaging than in other children. Family members who may want advanced imaging in order to make the correct diagnosis need to be educated that imaging involves tradeoffs and while potentially beneficial, imaging using ionizing radiation is associated with a small but real risk of future cancer. While some imaging cannot be reduced, further research may help identify areas that may be eligible for additional image reduction or alternative imaging methods in clinical practice.

### Strengths and limitations

The strengths of this study include its large sample size, accurate identification of children with Down syndrome, and detailed quantification of imaging over time. The proportion of children with Down syndrome (1/1400) is lower in our study compared to the national average of 1/700 births. This may be due to differences in maternal age and prenatal screening in cohort members or due to the six-month enrollment requirement, which may have under captured some Down syndrome cases in children enrolled for a short period of time. Nonetheless, this is unlikely to impact the characterization of imaging use in children with Down syndrome.

We were cautious not to overcount imaging by only including a maximum of one examination per modality and anatomic area per child and day, but as a limitation, we may have omitted real exams while trying to remove potential duplicates (e.g., two chest radiography exams on the same day would be counted as one). True imaging rates may be even higher than we report, though we do not expect a differential impact by whether children had Down syndrome. Our study population consisted of children with health insurance which may limit the generalizability of the results, though we were able to include children on Medicaid and adjust for Medicaid insurance in the analysis. Insurance coverage, provider type, and socioeconomic status differences between children with and without Down syndrome may also influence factors that determine how often imaging will occur or what type of imaging a child may receive.

## Conclusion

In this large, contemporary U.S. cohort study, children with Down syndrome experienced more imaging than other children. Notably, they received more imaging with modalities that use relatively higher amounts of ionizing radiation such as CT and angiography and at younger ages when they are most sensitive to ionizing radiation. While pediatric imaging using ionizing radiation has generally decreased in the last decade, imaging use increased in children with Down syndrome. Given the high exposure to imaging with radiation in children with Down Syndrome, especially at a very young age, future research should evaluate whether imaging with ultrasound and MRI that do not use ionizing radiation could replace some of the imaging with CT and angiography to reduce the risk of radiation-induced cancer.

## Abbreviations

CI: confidence interval
py: person-years
RR: relative rate
Q1: first quartile
Q3: third quartile
