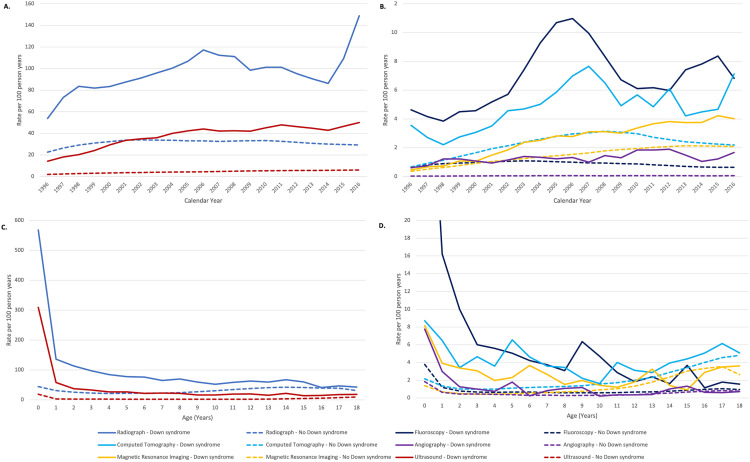# Correction: Medical imaging utilization and associated radiation exposure in children with down syndrome

**DOI:** 10.1371/journal.pone.0333514

**Published:** 2025-09-29

**Authors:** The PLOS One Staff

In Fig 1, the legend that describe the line color and shape of a, b, c, and d is missing. Please see the correct Fig 1 here. The publisher apologizes for the error.

**Fig 1:**